# The impact of digital finance on young People’s health insurance participation decisions in China

**DOI:** 10.3389/fpubh.2024.1370936

**Published:** 2024-07-01

**Authors:** Mengran Chai, Lin Wu

**Affiliations:** School of Sociology, Wuhan University, Wuhan, China

**Keywords:** digital finance, health insurance, social welfare, participation decision, rational choice

## Abstract

**Background:**

The digital finance era has reshaped young people’s lifestyles, risk perceptions and insurance participation decisions. Modern youth have to constantly seek for rational social security support and construct individual protection barriers to adapt to new lifestyles and social structures. China’s multi-tiered, universal health insurance system is urgently needed to satisfy young people’s flexible needs and rational decision-making.

**Methods:**

Based on the micro data from 2011 ~ 2021 Chinese General Social Survey (CGSS), this paper uses macro data from Digital Inclusive Finance Index (DIFI) matching to construct probit and m-logit model to assess the impact of the development of digital finance on Chinese youth whether or not to participate in health insurance, and how they choose the concrete type of health insurance.

**Results:**

(1) Baseline regression results shows that digital finance has a significant positive effect on Chinese youth’s health insurance participation decisions, and has different effects on choices within health insurance types. Strong support for the conclusions is provided by endogeneity and robustness tests. (2) The results of the heterogeneity analysis reveal that the marginal effect of digital finance on young people’s health insurance decisions shows urban–rural differences, divergence in levels of self-rated health. (3) The mechanism analysis results suggest that there are two mechanism paths of digital finance on youth health insurance decisions: the household income effect and the subjective well-being effect, and two moderating effects: employment type and family structure.

**Conclusion:**

Highlighting the positive value that digital finance brings to the perception of youth insurance participation and the construction of social security systems, it needs to pay close attention to the dynamic changes in employment security and family structure through data, and explore the socio-psychological fluctuation and demand for social security among modern youth. To provide a way forward to achieve the integration of the health insurance system in China and solve the current problem of health insurance equity.

## 1 Introduction

**Figure 1:**
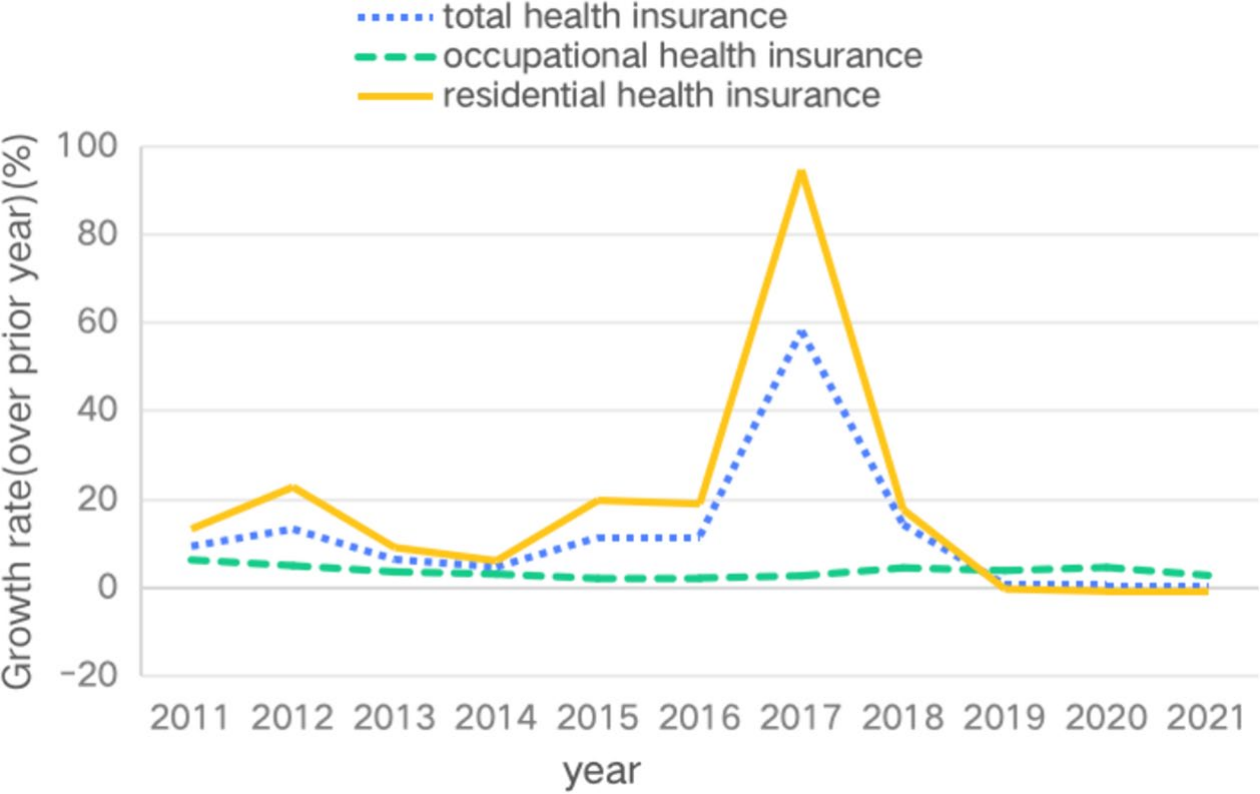
Growth rate of basic health insurance in China (2011 ~ 2021).

For China, with its large population, a more complete, accurate and convenient health insurance system is of great value in improving the accessibility of medical services, promoting the rational allocation of medical resources and fostering social stability. Data from the National Health Security Bureau shows that “by the end of 2022, the number of people covered by basic health insurance nationwide was 134,592,000, with a stable participation rate at over 95%” (1). However the slowdown in the growth rate of basic health insurance over the past decade, and especially the reversal in resident health insurance over the past 3 years (see Figure 1). There may be a delayed structural flaw in the health insurance system that does not sufficiently motivate the opportunity and willingness of residents to participate in insurance. According to the seventh national census, China’s total population has exceeded 1.41 billion (2), with young people aged 14–35 accounting for about 28.4% of the total population (3). Along with profound changes in economic development, consumption patterns, investment channels and types of employment, young people have experienced enormous changes in their social structure, lifestyles, risk perceptions and cultural attitudes. Influenced by the mixed welfare consciousness, the youth group has the highest willingness to participate in health insurance among the social security system (4), but influenced by the macro-environment, economic foundation and individual life course, the health insurance participation behaviour of the youth group in the digital era has the characteristic of prudent and rational choice. Firstly, today’s young people are more rational in their perception of insurance participation, paying attention to their physical and mental health, economic risks, wealth distribution and medical protection with a longer-term perspective and rational thinking, and they have an earlier recognition of and demand for health insurance, seeking more reasonable and diversified risk protection support. Secondly, there is a dilemma in the opportunity for young people to participate in insurance. Due to the characteristics of China’s social health insurance system, which is legally required for employers to provide social health insurance for their employees and voluntary for individual residents, the participation rate of China’s health insurance is closely related to the employment rate. However, according to data from the National Bureau of Statistics, the unemployment rate of Chinese youth in recent years is between 10 and 20% (5), and the unemployment situation reduces the chances of young people being forced by their employers to take out social health insurance, and limited to the financial constraints and prudent measurement of costs and benefits, it reduces the subjective motivation of young people to participate in social health insurance voluntarily. Finally, young people’s demand for insurance is flexible. With social transformation and the development of internet technology, the proportion of flexible workers among the youth group has increased. Data from the 2022 National Comprehensive Survey on the State of Youth Development shows that the participation rate of basic health insurance for young workers working in new formats on internet platforms is only 60%. The structure of health insurance for the youth group still faces the risk of “reverse selection” and the dilemma of differentiated needs.

Digital technology has injected kinetic advantages into economic transformation and high-quality development. China’s digital finance has shown strong development momentum, and its unique characteristics of low cost, wide coverage and convenient accessibility have had a broad and far-reaching impact on socio-economic sectors, which has played a multidimensional role in promoting the development of health insurance. Firstly, digital finance has the effect of alleviating financial constraints. Digital finance can stimulate economic development and individual income levels; digital finance platforms offering instalment, credit and other payment methods can ease financial pressure on young people; young people begin to pay attention to their health care and risk protection as their disposable income increases. Secondly, digital finance enhances the opportunity effect. On the one hand, mobile payment and online services can simplify the process of buying health insurance and making claims, provide more personalised insurance solutions, and offer more diversified options for young people to choose health insurance; on the other hand, digital finance can create more jobs and increase opportunities for young people to passively participate in insurance. Thirdly, digital finance provides an information dissemination platform and accessible channels. Online communication and precise information provision provide a practical path for health insurance to reach all social strata in a comprehensive way, and at the same time, it can improve the risk management and pricing strategy of the insurance service body, so as to expand the coverage of health insurance among youth group. Accordingly, in the context of digital finance, the transformation of the macro social security system and the risk perception and rational choice of the micro youth group are social issues that require urgent attention. This paper aims to start from the cognition and needs of the youth group, use rational choice theory as the analytical framework, combine macro and micro data, conduct an observational study of the youth’s choice tendency in the field of health insurance in the digital financial era, and explore the potential mechanism in depth.

## 2 Theoretical background

As an important part of social security, health insurance is a “stabiliser” of social development and an “enabler” of the economy. For individuals, participation in health insurance is an important aspect of the distribution of individual and family wealth, and is a rational choice based on individual and family risk expectations and investment of capital costs (6). Particularly for young people with limited earning power and asset accumulation, the value of insurance participation reflects young people’s anxiety about potential risks and their desire for a better life. With the deep development of the application of digital technology and the profound changes brought about by the digital economy, the inclusive feature of digital finance is to provide comprehensive financial services at an affordable cost to social groups from all classes (7). At the macro level, it brings new opportunities and challenges for the construction and development of the social security system, and at the micro level it also opens up new avenues and dilemmas for the income and expenditure of assets and the risk protection of young people. The study of the value of digital finance for social security and insurance systems has become an important topic in recent years.

Digital finance has strengthened the instrumental function. First, in terms of the derivative characteristics of the instrument function, digital finance has built a convenient transaction platform for the main body of insurance services, promoted the in-depth development and use of financial technology by insurance enterprises, expanded the marketing channels (8), and greatly increased the cognitive attention of potential customers, the willingness of contact and the interaction of insured. Second, in terms of the cost attributes of the instrument function, digital finance significantly reduces the cost of time and effort in searching, comparing and processing information for customers’ insurance decisions (9). Meanwhile, the reduction in indirect participation costs of digital finance enables customers to reap more financial benefits than they would through traditional channels. Third, in terms of the uptake characteristics of the instrumental function, the strong availability of digital finance is particularly evident among the youth population. The temporal and spatial advantages, risk characteristics and transaction modes of digital finance are better suited to youth’s digital literacy, risk appetite, consumption information and long-term stickiness of insurance coverage, increasing their subjective willingness to take out health insurance.

Digital finance tends to be more practical at informing. First, in terms of the risk perception of the information function, compared with traditional finance, digital finance has lower operating costs, can better overcome the weakness of limited information (10), and resolve the structural risk that may arise from information asymmetry (11). Second, in terms of interactive spillovers from information functions, digital finance enhances information flows and facilitates more regulated social interactions, which helps young people generate stronger endogenous motivation to engage in self-interested health insurance (12). Third, in terms of the benefits of the information function, digital finance can provide richer information about product choices, expand the supply of financial services (13), and overcome the disadvantages of past financial exclusion. Accordingly, the research hypothesis 1 is formulated.

*Hypothesis 1*: Digital finance can have a significant impact on youth decisions about health insurance.

As a vital component of the digital economy, digital finance has facilitated the enrichment and flexible transformation of forms of employment for young people. Digital finance expands the employment structure and job opportunities in the financial services sector, enhances young people’s digital work skills and provides more flexible employment opportunities. However, changes in labour relations brought about by new forms of employment have led to multidimensional risk taking and social rights claims by young workers (14), as well as the decoupling and absence of social security rights. A new type of worker, represented by ‘net contract workers’, faces the dilemma of high exposure to occupational health risks and ambiguous employment relationships. Meanwhile, the new self-employed groups represented by “digital nomads” face the dilemma of intellectualised and elastic group characteristics with rationalised and diversified risk protection needs (15). Therefore, the employment patterns of the growing proportion of youth with flexible employment in the context of the digital economy have an impact on health risk perceptions, health insurance awareness and decisions. Moreover, in the context of changing social attitudes and adjustments in fertility policies, family structure affects family risk perceptions and financial asset allocation. The number of children and parental demand for commercial health insurance in young families with limited wealth have a negative inhibiting effect (16), while family mobility has a positive facilitating effect on young people’s participation in basic health insurance (17). So, family structure may have influence on how digital financial affects youth health insurance decisions, to formulate the research hypothesis 2.

*Hypothesis 2*: Moderating effects of employment type and family structure in the impact of digital financial on youth’s health insurance decisions.

Income is an important variable in studies of digital finance, social security and medical insurance. Digital finance in general has a ‘digital dividend’ effect (18, 19), reducing income disparities and bridging the ‘digital divide’ (20). For individuals, the increase in earning capacity and income levels, the increased awareness of self-protection and the costs of risk protection (21), the change in risk preferences and the fear of returning to poverty (22, 23), and the pursuit of individual and structural optimisation within the framework of rational choice (1) will facilitate participation in or supplementation with health insurance (24). In addition, Wang et al. (25) found a mediating effect mechanism for the income effect in the study exploring the impact of digital finance on social security from a macro perspective. For the youth, a measure of household income is an important basis for allocation decisions, so household income may be a mechanism variable influencing the youth’s health insurance decisions. Then, subjective well-being is also a key variable in the study of digital financial inclusion attributes and social security systems. Digital finance increases the availability of financial services and low-risk financial participation for residents, and the low-threshold experience of sharing the benefits of economic development increases residents’ perceptions of well-being (26). Digital finance provides opportunities and financial support for employment and entrepreneurship (27), and promotes efficiency and equity. Mobile payments and data services can better meet the consumption needs of the population in terms of time and transaction costs, reducing the pressure of economic interaction and institutional exclusion (28). Moreover, subjective positive attitudes have a positive impact on residents’ insurance participation (29), so subjective well-being encourages the optimisation of household asset allocation and increases insurance participation (4). Thus, subjective well-being may be another mechanism variable. Accordingly, the research hypothesis 3 and 4 were formulated.

*Hypothesis 3*: Household income as a mechanism variable for the impact of digital finance on the health insurance decisions of young people.

*Hypothesis 4*: Subjective well-being as a mechanism variable for the impact of digital finance on the health insurance decisions of young people.

**Figure 2:**
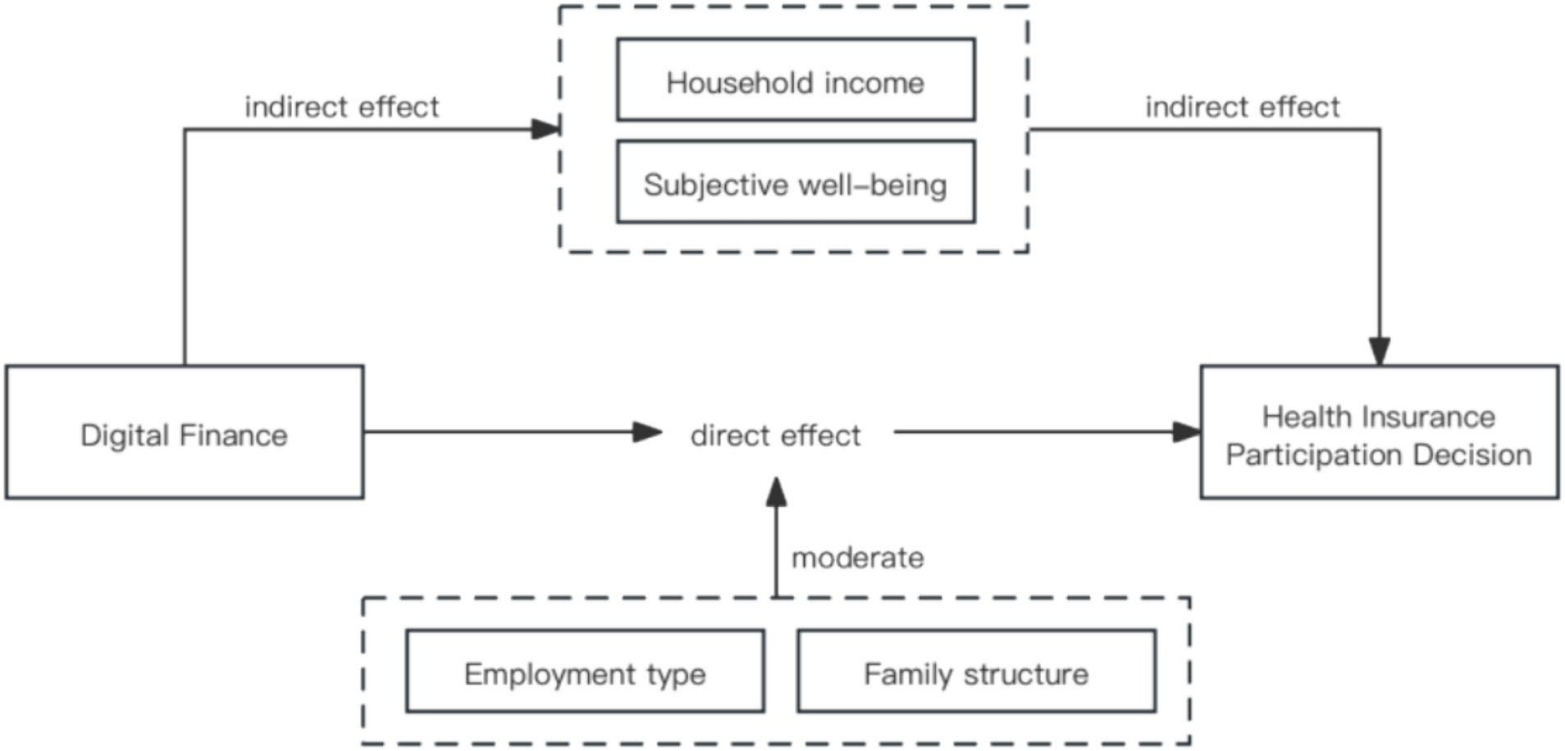
Research model.

In summary, the existing literature on the impact of digital finance and health insurance, mostly focused on the macro perspective of the positive or negative impact of digital finance on the construction of social security, while the youth group as the backbone of society, to explore the impact of digital finance on the cognitive changes and behavioural choices of the youth of health insurance, the improvement of multi-level social security system, alleviate the stratification of the welfare of youth is of practical significance and important value (Figure 2).

## 3 Materials and methods

### 3.1 Data sources

The micro-data come mainly from the Chinese General Social Survey (CGSS), which was launched in 2003 and is the earliest nationwide, comprehensive and continuous scientific survey in China. For each year of the survey, individual sampling is conducted using a multi-stage stratified PPS random sampling method. It covers 28 provinces (excluding Xinjiang, Tibet, Hong Kong, Macau and Taiwan) and has high representativeness, reliability and coverage. Specifically, given the development of the digital economy and the accessibility of key variables, six periods of CGSS data from 2011, 2013, 2015, 2017, 2018 and 2021 were used, focusing on the youth cohort aged 18–35. This resulted in a valid sample of 10,524 after removing missing values for key variables and performing data cleaning. The macro-data on the development of digital economy come from the Peking University Digital Inclusive Finance Index (2011–2021) published by the Centre for Digital Finance Research at Peking University, matched by the year of interview and region of the respondents in the micro-data sample.

### 3.2 Variable descriptions

#### 3.2.1 Dependent variable: health insurance participation decisions of young people

That is, whether or not the youth have health insurance, which in China has a distinction between basic health insurance and commercial health insurance. The questionnaire asked ‘Are you currently enrolled in any of the following social security programme?’ The responses for health insurance included ‘basic health insurance’ and ‘commercial health insurance’. After identification, a binary variable for ‘participation in health insurance’ (0 = non-participation, 1 = participation) and a multivariate categorical variable for ‘participation in basic health insurance versus commercial health insurance’ (0 = neither-participation, 1 = only basic health insurance, 2 = only commercial health insurance, 3 = both-participation) were created.

#### 3.2.2 Independent variable: development of digital finance

Digital finance generally refers to a new financial model developed by Internet enterprises using digital technology. Following the methodology of Xie et al. (30), the digital inclusive finance index for the relevant years in the Peking University Digital Inclusive Finance Index (2011–2021) published by the Digital Finance Research Centre of Peking University was used to measure the level of digital finance development. The indicator system of Digital Inclusive Finance Index, which is calculated using the Analytic Hierarchy Process, includes three dimensions: the breadth of digital finance coverage, the depth of digital finance use, and the degree of digitisation of inclusive finance, with 33 specific indicators.

#### 3.2.3 Control variables

Based on existing literature and data availability, three dimensions of control variables were included: individual characteristics, family characteristics and subjective value judgements. Individual characteristics include gender, age, education, hukou, and employment. Family characteristics including marriage, children and *per capita* household income. Happiness and subjective social stratum are subjective value judgements (31).

#### 3.2.4 Mechanism variables

In order to better understand the potential pathways through which digital financial development affects youth health insurance decisions, this paper incorporates two mediating mechanism variables: the household income effect and the subjective well-being effect. As the dependent variable is categorical, the mediator variable needs to be treated as categorical. For the household income effect, the median of the logarithm of total household income is calculated, with greater than the median identified as ‘high household income’ and less than or equal to the median identified as ‘low household income’. The subjective well-being effect uses happiness items and identifies binary variables, identifying responses of ‘very happy’ and ‘relatively happy’ as ‘higher happiness’ and responses of ‘medium’, ‘relatively unhappy’ and ‘very unhappy’ as ‘lower happiness’. Table 1 shows the variables defined and the descriptive statistical analyses.

**Table 1:** Descriptive statistics of samples.

**Variables**	**Mean**	**S.D**	**Min**	**Max**	**N**
*Dependent* var*iables: health insurance participation decisions*					
Participation in health insurance	0.885	0.318	0.0	1.0	10,524
Participation in basic versus commercial health insurance	1.190	0.816	0.0	3.0	10,524
*Independent Variable: development of digital finance*					
Development of digital finance	236.445	95.737	18.3	445.4	10,524
*Control Variables*					
Gender	0.478	0.500	0.0	1.0	10,524
Age	27.779	4.930	18.0	35.0	10,524
Education	0.415	0.493	0.0	1.0	10,524
Hukou	0.387	0.487	0.0	1.0	10,524
Employment	0.709	0.454	0.0	1.0	10,524
Marriage	0.600	0.490	0.0	1.0	10,524
Children	0.543	0.498	0.0	1.0	10,524
*Per capita* household income	9.975	1.147	1.4	16.0	10,524
Happiness	2.074	0.765	1.0	5.0	10,524
Subjective social stratum	3.273	0.670	1.0	5.0	10,524
*Mechanism Variables*					
Household income	0.495	0.500	0.0	1.0	10,524
Subjective well-being	0.806	0.395	0.0	1.0	10,524

### 3.3 Empirical strategy

First, since the dependent variable ‘participation in health insurance’ is a binary categorical variable, the probit model is used, where *insurance* represents the health insurance decision, *DIFI* represents the level of digital financial development, *β* is the coefficient that this paper focuses on, and *X_it_* represents a set of control variables, with the specific formulas as equation (1):


(1) $$\mathrm{Probit}\left(\mathrm{insuranc}e_{\mathrm{it}}\right)=\Phi \left(\alpha +\mathrm{\beta DIF}I_{\mathrm{it}}+\gamma X_{\mathrm{it}}\right)$$

Second, since the dependent variable ‘Participation in basic health insurance versus commercial health insurance’ is a four-categorical variable, the M-logit model regression model is constructed, in which *insurance1_it_* represents the type of health insurance decision, *j* is the value of the categorical variable, *X_it_*’ is a set of parameter vectors, and *DIFI* is the level of digital financial development, the specific formulas are as equation (2):


(2) $$\begin{array}{l} \mathrm{Prob}\left(\mathrm{Insurance}1_{\mathrm{it}}=j\;|\;X_{\mathrm{it}}\right)=\frac{\exp\left({X_{\mathrm{it}}}^{\prime }\;\mathrm{DIFI}\right)}{\sum_{j=1}^{J}\exp\left({X_{\mathrm{it}}}^{\prime }\;\mathrm{DIFI}\right)},\\ j=0,1,2,3 \end{array}$$

Using the dependent variable = 1(only participate basic health insurance) as the base group, the Odds Ratio of each option relative to the base group can be expressed as equation (3):


(3) $$\ln\frac{P_{ijt}}{P_{ikt}}={X_{\mathrm{it}}}^{\prime }\left(DIFI_{jt}-DIFI_{kt}\right),k=1$$

## 4 Results

### 4.1 Baseline regression analysis

The regression results based on the empirical strategy are presented in Table 2. First, the impact of digital financial development on the youth group’s choice to participate in health insurance is shown in Table 2 column (1), and is statistically significant at the 1% level: for every standard deviation increase in digital financial development, the probability of the youth group participating in health insurance increases by 0.3%. This indicates that the development of digital finance in terms of depth, breadth and digitisation has a significant positive impact on the youth group’s health awareness and choice of health insurance, initially supporting Hypothesis 1. Second, column (2) of Table 2 shows the differentiation of young people’s choice of the specific type of health insurance. The results are all significant at the 1% level and Hypothesis 1 is further verified. Specifically, digital finance significantly reduces the probability of ‘neither-participation’ and ‘commercial health insurance only’ among young people, and significantly increases the probability of ‘both-participation’, compared to the ‘basic health insurance only’ group. This has a positive impact on the construction of the social security system, which can improve the sense of happiness, security and fairness of Chinese youth (32), enrich their dimension of medical insurance, and enhance their expected health and sense of economic security, thus improving the economic health security of young people and their confidence in future development.

**Table 2:** Baseline regression results.

	(1) Probit	(2) M-logit(based on group ‘basic health insurance only’)		
	0 = non-participation, 1 = participation	‘Neither-participation’/'basic health insurance only’	‘Commercial health insurance only’/'basic health insurance only’	‘Both-participation’/'basic health insurance only’
DIFI	0.003***	−0.005***	−0.006***	0.004***
	(14.51)	(−14.01)	(−6.99)	(8.65)
Gender	0.047	−0.084	0.178	0.057
	(1.32)	(−1.23)	(1.24)	(0.90)
Age	0.011**	−0.016*	−0.013	0.018**
	(2.27)	(−1.80)	(−0.64)	(2.04)
Education	0.399***	−0.677***	0.160	0.592***
	(9.36)	(−8.20)	(0.96)	(7.98)
Hukou	−0.049	0.155**	0.468***	0.334***
	(−1.21)	(2.02)	(2.98)	(4.84)
Employment	0.153***	−0.309***	−0.649***	−0.009
	(3.79)	(−4.04)	(−3.97)	(−0.12)
Marriage	0.161***	−0.333***	−0.531**	−0.136
	(2.73)	(−2.93)	(−2.11)	(−1.33)
Children	0.124**	−0.191	0.715***	0.237**
	(2.05)	(−1.64)	(2.81)	(2.29)
*Per capita* household income	−0.008	0.077**	0.424***	0.377***
	(−0.39)	(1.99)	(5.17)	(10.34)
Happiness	−0.075***	0.138***	0.108	−0.132***
	(−3.37)	(3.33)	(1.16)	(−2.96)
Subjective social stratum	−0.142***	0.242***	−0.043	−0.246***
	(−5.23)	(4.66)	(−0.39)	(−4.93)
Provinces	Yes	Yes	Yes	Yes
Cons	0.558**	−1.305**	−5.966***	−6.205***
	(2.01)	(−2.42)	(−5.27)	(−12.36)
N	10,524	10,524		
Pseudo R2	0.0994	0.1157		

*p < 0.05; **p < 0.01; ***p < 0.001.

Among the individual characteristics of the control variables, age has some influence on the decision to participate in health insurance, that is the probability of participating in health insurance increases by 1.1% for every one-year increase in the age. Compared with the ‘basic health insurance only’ group, the probability of ‘neither-participation’ decreases by 1.6% and that of ‘both-participation’ increases by 1.8%, while there is no significant effect for ‘commercial health insurance only’. This means that individuals’ perceptions of health risk, potential medical expenditure and life stage fluctuate with age, triggering individuals’ concerns about health insurance and generating rational expectations and decisions. Young people with higher education are about 40% more likely to have health insurance than those without. In comparison with the ‘basic health insurance only’ group, the probability of ‘neither-participation’ among highly educated people decreases by 67.7% and that of ‘both-participation’ increases by 59.2%, consistent with previous research (33). The difference between urban and rural participation in health insurance is no longer apparent, but there is a significant positive increase in probability of making different decisions for urban youth compared to the ‘basic health insurance only’ group. Perhaps this is because the proportion of flexible workers among urban youth is increasing, as is the demand for more diversified and complex health insurance options. Young people with a job may have a 15.3% higher probability of participation in health insurance. In contrast to the ‘basic health insurance only’ group, young people with a job are less likely to be ‘neither-participation’ (30.9%) and ‘commercial health insurance only’ (64.9%), while there is no significant effect on ‘both participation’. This is related to the mandatory nature of basic health insurance for employers in China.

Among the family characteristics, marriage and children have a significant impact on the youth’s decision to participate in health insurance, as family structure, risk perception and risk appetite will lead them to consider health insurance participation rationally. Household income *per capita* does not have a significant effect on whether people participate in health insurance, but it does have a significant effect on the choice of concrete types. There are differences in health risk perception, insurance awareness and risk-taking ability between people with different income levels (34). In the dimension of subjective value judgements, happiness and subjective social stratum reflect young people’s attitudes to their position in the social structure, which influences risk preferences and wealth decisions. The enhancement of the individual’s subjective value judgement increases the odds of buying health insurance, which is also in accordance with the previous study (35).

### 4.2 Endogeneity and robustness analysis

To overcome potential endogeneity issues and to further examine the robustness of the estimation results, this paper uses five methods: using the instrumental variable method, replacing the model, replacing the independent variables, eliminating extreme values and controlling potential time trend. Table 3 shows the results. Firstly, this paper uses the spherical-distance from each province to Hangzhou as an instrumental variable for endogeneity test of digital financial development, and the results are shown in columns (1) and (2) of Table 3. According to Guo et al. (36), digital finance in China originated from Alipay established in Hangzhou, and digital finance has an obvious spatial agglomeration effect, so the spherical-distance from Hangzhou and the development of digital finance have a significant correlation; however, the spherical-distance from Hangzhou does not directly affect the decision of the youth to participate in health insurance, thus fulfilling the exogeneity requirement. The VIF of the important variables is less than 5, which means there is no multicollinearity problem, and the estimates of the first-stage regression are significant at the 1% level and the F-statistic value is greater than 10, which rejects the initial hypothesis of a weak instrumental variable. In the second-stage, the estimated value is still significant at the 1% level, the estimated coefficient is 0.008, indicating that the previous regression results are robust and further support Hypothesis 1.

**Table 3:** Endogeneity and robustness results.

	Instrumental variable		Replace model	Change independent variable	Financial development control	Remove extreme values	Time trend control
	(1) First-stage	(2) Second-stage	(3) Logit-model	(4) Financial development	(5) Financial development as a control	(6) Remove municipalities	(7) Yearly trend
DIFI		0.008***	0.005***	0.555***	0.003***	0.003***	0.006***
		(4.62)	(14.76)	(11.71)	(8.46)	(13.00)	(6.82)
Spherical-distance	−0.037***						
	(−12.33)						
Financial development					0.070		
					(0.95)		
Yearly trend							−0.121***
							(−3.82)
Controls	Yes	Yes	Yes	Yes	Yes	Yes	Yes
Provinces	Yes	Yes	Yes	Yes	Yes	Yes	Yes
Constant	−46.93***	0.839*	0.935*	−2.160***	0.228	1.712***	243.418***
	(−3.47)	(2.51)	(1.76)	(−6.17)	(0.51)	(5.78)	(3.83)
N	10,524		10,524	10,524	10,524	8,556	10,524
Wald-test P	0.0026						
Pseudo R2			0.1009	0.0898	0.0995	0.0908	0.1013

*p < 0.05; **p < 0.01; ***p < 0.001.

Secondly, since the dependent variable ‘participation in health insurance’ is a binary categorical variable, the logit-model is used to replace probit-model in baseline regression for the robustness test. The results are shown in column (3) of Table 3. The results are still significant at the 1% level and the coefficients are not very different from those in the probit-model.

Thirdly, referring to Jiang et al. (37) and Wang et al. (25), to avoid the possible endogeneity of the independent variables with other control variables, the ratio of total deposits and loans to GDP is used as an indicator of the level of financial development to replace DIFI, and the results are shown in column (4) of Table 3. Consistent with the results using DIFI as the independent variable, the results are still significant at the 1% level.

Fourthly, by using the ratio of total deposits and loans to GDP as an indicator of the level of financial development to replace DIFI in column (4) of Table 3, there might be a risk of an alternative causality whereby the level of financial development facilitates both the development of DIFI and the youth health insurance decision. Considering the above, financial development was included again as a control variable and the results are shown in column (5) of Table 3. The results show that DIFI remains significant at the 1% level for the youth health insurance decision, but the level of financial development (the ratio of total deposits and loans to GDP) is not significant (*p* = 0.344). The above results address potential causality risks and more rigorously validate the robustness of the findings.

Then, the level of economic development in various regions may affect the growth of digital finance, with reference to Qian et al. (38), the sample of four municipalities is removed, and the results are presented in column (6) of Table 3. There has been no noticeable change in the significance and coefficients, indicating that the results are robust.

Finally, considering the effect of the potential time trend, following Li et al. (39) and Wang et al. (40), the time trend term is included in the regression and the results are as in column (7) of Table 3. After controlling for the potential time trend factor, the results remain significant at the 1% level and the correlation coefficients do not change significantly, thus further validating the theoretical assumptions of the paper and the robustness of the data results.

### 4.3 Heterogeneity analysis

Digital finance can expand health information channels, improve access to medical services and ease capital financing constraints, while individuals’ perception of health risk and ability to bear risk will influence their health insurance participation decisions. At the macro dimension, there exist urban–rural differences in the development of digital economy. Urban and rural youth differ in the aspects of wealth distribution, life mobility, physical and mental stress, and access to information that shape health insurance decision behaviours. Table 4 shows that digital finance has a significant impact on the choice of type of health insurance for both urban and rural youth, but the marginal effect is different. Using ‘basic health insurance only’ as a baseline, digital finance has a stronger effect on reducing the probability of urban youth ‘neither-participation’ and ‘commercial health insurance only’. In urban areas, greater density of health insurance information can raise youth’s awareness of health risks and ‘poverty through illness and return to poverty’, and increase the acceptance rate and motivation to participate in basic health insurance (41). While rural youth are more mobile and the dissolution of household registration and identity leads to a more rigorous need to budget disposable income. Compared to the relatively long-term health risks and easing of budget constraints generated by digital finance, they have a stronger tendency to maintain an acceptable level of health protection, resources and balance the existing channel to income generation.

**Table 4:** Heterogeneity results: urban–rural.

	Urban youth			Rural youth		
	‘Neither-participation’	‘Commercial health insurance only’	‘Both-participation’	‘Neither-participation’	‘Commercial health insurance only’	‘Both-participation’
DIFI	−0.007***	−0.007***	0.004***	−0.004***	−0.004***	0.003***
	(−11.30)	(−6.84)	(6.76)	(−8.98)	(−2.90)	(5.15)
Controls	Yes	Yes	Yes	Yes	Yes	Yes
Provinces	Yes	Yes	Yes	Yes	Yes	Yes
Cons	−0.967	−4.180***	−5.516***	−0.822	−6.872***	−6.487***
	(−1.03)	(−2.61)	(−7.61)	(−1.18)	(−4.11)	(−8.91)
N	4,077			6,447		
Pseudo R2	0.1302			0.1043		

1. Use ‘basic health insurance only’ as a baseline; 2. *p < 0.05; **p < 0.01; ***p < 0.001.

In the micro dimension, items from the individual self-rated health section are used to identify responses of ‘very good’, ‘relatively good’ and ‘good’ as good self-rated health and responses of ‘relatively poor’ and ‘very poor’ as poor self-rated health, and the results are shown in Table 5. Compared to ‘basic health insurance only’, the marginal effect reduction of ‘neither-participation’ did not differ by self-rated health. However, the marginal effect of ‘commercial health insurance only’ is significantly reduced for youth with good self-rated health, while it is not significant for youth with poor self-rated health. This may be limited to the crowding-out effect of basic commercial health insurance (42). Individuals with good self-rated health have lower expectations of their own health risks and tend to accept the basic protection offered by basic health insurance, which also suggests that digital finance is conducive to mitigating the risk of adverse selection in basic health insurance (43). Meanwhile, the chances of ‘both-participation’ are higher for individuals with poor self-rated health than for those with good self-rated health. A possible reason is that people with poor self-rated health have a lower threshold for anticipated health risks and greater rational anxiety about self- and family-protection (44). Digital finance has increased the choice and protection dimension of health insurance, improved the convenience of insurance services and the availability of potential funds, increasing the ability of youth to manage health risks. It can offset the pressure on public funding from adverse selection in social health insurance.

**Table 5:** Heterogeneity results: self-rated health.

	Good self-rated health			Poor self-rated health		
	‘Neither-participation’	‘Commercial health insurance only’	‘Both-participation’	‘Neither-participation’	‘Commercial health insurance only’	‘Both-participation’
DIFI	−0.005***	−0.005***	0.003***	−0.005***	−0.006	0.008***
	(−12.42)	(−6.34)	(7.43)	(−4.16)	(−1.40)	(4.45)
Controls	Yes	Yes	Yes	Yes	Yes	Yes
Provinces	Yes	Yes	Yes	Yes	Yes	Yes
Cons	−1.181**	−6.082***	−5.935***	−3.219	−6.591	−11.484***
	(−2.08)	(−5.15)	(−11.55)	(−1.63)	(−1.31)	(−4.09)
N	9,844			680		
Pseudo R2	0.1099			0.2845		

1. Use ‘basic health insurance only’ as a baseline; 2. *p < 0.05; **p < 0.01; ***p < 0.001.

### 4.4 Mechanism analysis

**Figure 3:**
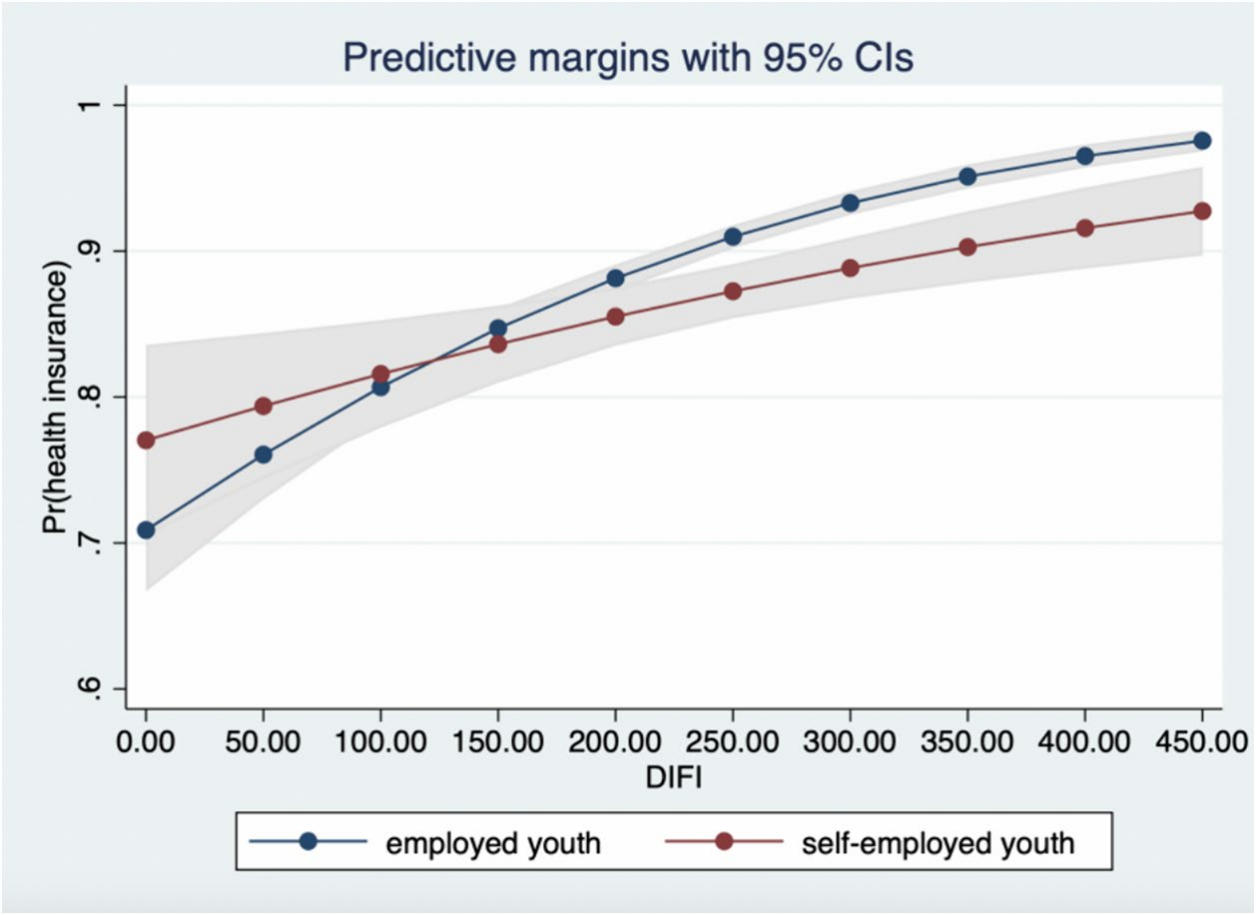
The moderating effect of employment type.

**Figure 4:**
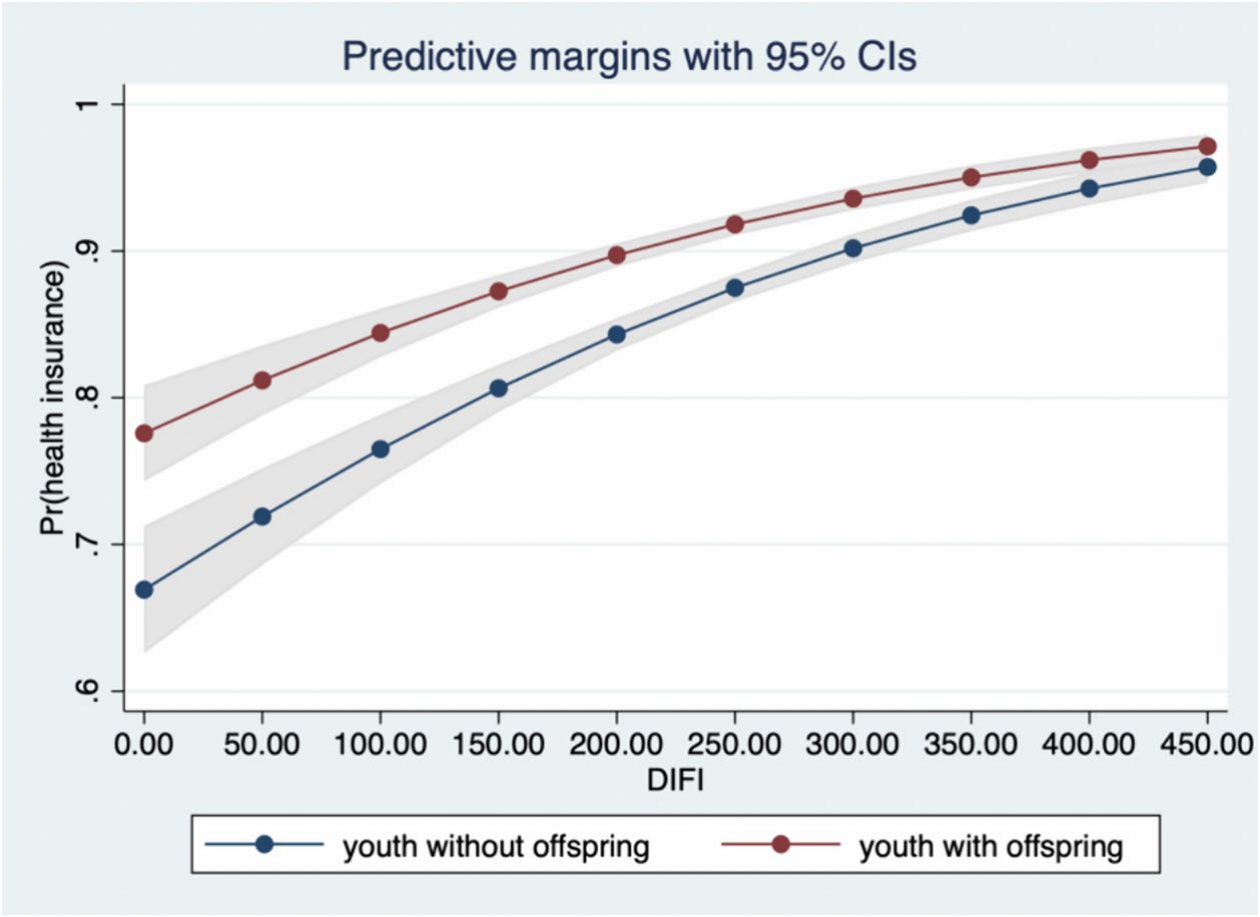
The moderating effect of family structure.

Hypothesis 2 of this paper proposes the moderating effect of employment type and family structure, with reference to Nowell’s (45) three-stage testing method, the moderating effect of the non-linear model is conducted by using the interaction term between explanatory variables and moderating variables. Figure 3 visually shows that the moderating effect of employment type, at the beginning of the development of digital finance, the effect is relatively overlapping between groups, but diverges after the digital finance index reaches 200, and the marginal effect of employed youth is stronger than that of self-employed youth, confirming Hypothesis 2. Figure 4 illustrates the moderating effect of family structure. As the development of digital finance deepens, the tendency of youth with and without offspring to participate in health insurance becomes more convergent from differentiated, supporting Hypothesis 2.

Based on the aforementioned theoretical mechanism analysis, this paper proposes two mechanism paths for the household income effect and the subjective well-being effect, respectively, and empirically tests them using the three-step test method. The results are shown in Table 6. Columns (1) (2) (3) show the results of the household income effect, and the mechanism effect is significant at the 1% level, confirming Hypothesis 3. This demonstrates that digital finance in general can stimulate the enhancement of young families’ incomes, shape the dimensions of health risk perception, family investment preferences, self-protection cost inputs and family risk-bearing capacity, and thus influence the propensity to make health insurance decisions. Columns (4) (5) (6) are the results of the subjective well-being effect, showing that the effect of the mediating mechanism is significant at the 1% level, proving Hypothesis 4. Digital finance enhances youth’s subjective well-being in the dimensions of economic development, consumption leap, and quality of life, and that youth’s evaluative dimensions of perceived future expected risk and comprehensive cognition of their own social structure provide rationality for health insurance choice reference.

**Table 6:** Mechanism results: household income effect and subjective wellbeing effect.

	Household income effect			Subjective well-being effect		
	(1)	(2)	(3)	(4)	(5)	(6)
	Participation decision	Household income	Participation decision	Participation decision	Subjective well-being	Participation decision
DIFI	0.003***	0.004***	0.003***	0.003***	0.000**	0.003***
	(14.94)	(24.66)	(13.67)	(14.57)	(2.36)	(14.49)
Household income			0.138***			
			(3.36)			
Subjective well-being						0.145***
						(3.42)
Controls	Yes	Yes	Yes	Yes	Yes	Yes
Provinces	Yes	Yes	Yes	Yes	Yes	Yes
Constant	0.475***	1.299***	0.355**	0.487*	2.430***	0.303
	(2.72)	(8.50)	(1.99)	(1.76)	(10.27)	(1.07)
Pseudo R2	0.0993	0.2887	0.1008	0.0978	0.0735	0.0994
N	10,524			10,524		

*p < 0.05; **p < 0.01; ***p < 0.001.

## 5 Conclusion and discussion

Based on data from the 2011–2021 Chinese General Social Survey, this paper empirically analyses the impact of digital finance on health insurance decision-making among youth groups and explores in depth the potential mechanisms. The results of this paper show that digital financial development has a significant positive impact on youth groups’ participation in health insurance, and a significant impact on their choice of concrete type of health insurance. The use of instrumental variables, replacement model, replacement of independent variables and elimination of extreme values all produce a consistent result. The development of the digital economy, although in the short term on the social security system produces ambiguity in labour relations (46), institutional integration lag and other problems (47), and even the existence of a certain space of moral hazard and “system failure” (48). However, it is vital to highlight the positive value that the digital economy brings to the public perception and system construction of health insurance, to make full use of the tool and information functions of digital finance, and to provide a thought path for realising the integration of the health insurance system and solving the current problem of health insurance equity. In the macro-construction dimension, the digital platform can promote the expansion of high-quality medical resources and the balanced layout of the region, and through the digital “tentacles,” the multi-level and orderly goal construction of the social security system can be effectively achieved, gradually solving the social security system’s problem of solidifying social stratification, and enhancing the youth’s sense of identity and access to social status; moreover, the platform attribute of digital finance can improve the service utilisation rate and precision service of health insurance, and the direct or indirect data information feedback provides a realistic basis for the digital transformation of basic health insurance and the rich design of commercial health insurance, so as to provide popular, balanced and moderate health insurance options for young people in different life situations. In the micro-service dimension, digital finance can provide diversified information channels for modern health insurance, and increase health risk awareness and health insurance knowledge among young people. Digital finance can compensate for young people’s financial constraints and provide multiple payment methods under a rational analysis logic of the possible medical burden and the risk of “cost–benefit.” Concurrently, the spatial separation of digital platforms can eliminate the marketing exclusion of youth groups from commercial health insurance and provide time and space for diversified needs, rational analysis and personalised choices, which is beneficial to mitigate the adverse selection of basic health insurance and broaden the popularity of high-quality health insurance. In addition, youth growing up in the digital age pay more attention to information security and privacy protection. Therefore, health insurance services on digital financial platforms need to devote more attention to the security protection of users’ privacy, enhance young people’s dual social trust in digital financial platforms and health insurance, and protect the positive incentive effect of digital finance on young people’s health insurance decisions. Overall, digital finance has a positive effect on expanding the coverage of basic health insurance and strengthening the construction of the social security system, which can enhance the sense of happiness, security and equity among Chinese youth, thus improving their economic health security and confidence in future development.

This paper finds that the individual’s employment type and family structure have moderating effects. First, with economic and social transformation and the rapid development of the digital economy, new opportunities are emerging in terms of employment for youth groups, and the forms of employment are also complex and diverse. The awareness and ability to dominate the medical risk protection of full-time employees based on occupational health insurance is increasing, while the risk of medical exposure has increased for young people employed in odd jobs in the digital era, represented by delivery staff and online drivers, and they are more inclined to opt for health insurance to protect themselves and their families economically in the case of ambiguous coverage for work-related injuries and other risks coverage. In addition, digital economy era has generated a new type of self-employed, such as ‘digital nomads’, who are generally highly educated, flexible and able to objectively assess their own health risks and needs (15). Constrained by fluctuating incomes, a high proportion of insurance contributions and a high level of spatial mobility, young people with an insecure daily life and an insecure future have created the need to rely on insurance services for the construction of economic and class protection. However, the stable and definable health insurance system adapted to previous generations can no longer fully accommodate the differentiated needs of modern youth. Then, the evolutionary changes in social structure and social culture have reshaped self-perception, the concept of marriage and family, and they are constantly searching for rational social support and secure individual barriers to adapt to new lifestyles and family structures. In previous generations, having a family and raising children meant entering adulthood, and compared to single young people, they were more concerned with maintaining the family hierarchy and economic security, so they came into contact with and participated in insurance services at an earlier stage. Whereas modern youth in the digital economy era tend to pay more attention to individual life course and liberal social time (49). As the proportion of young people living alone, Dink families and other family structures increase, their future development and life protection, health care and other needs are at the forefront. The practices of this group of young people who try to ‘reverse the social clock’ need to take greater account of the potential risks of individual choices without disrupting the wealth and normal life of their parents, while digital finance could provide them with more appropriate information channels, freedom of choice and loosened financial constraints.

This paper reveals the mechanism path, which includes household income and subjective well-being. First, digital finance in general has the effect of a ‘digital dividend’ and the attribute of ‘universal benefit’. It provides information channels and leapfrogging opportunities for young people in terms of income distribution and wealth accumulation, which can increase their determination and willingness to participate in health insurance. Second, digital finance can balance efficiency and fairness, bridging the ‘digital divide’ and building convenient channels for mobile payments, financial literacy and market participation. Meanwhile it provides a possible way to improve the construction of the health insurance system, increase the density of information coverage, actively guide young people to recognise the differences in the protection dimensions and capabilities of health insurance. It pays close attention to the dynamic changes in employment security and family structure through data, and explores the socio-psychological fluctuation and demand for social security, so as to improve the support efficiency of health insurance provision and play the role of social stabiliser of the health insurance system.

Notably, based on the rational-choice perspective, this paper explores the behavioural decision-making tendency of youth’s health insurance in the course of digital financial development from the macro perspective of social structure and socio-psychological change using an empirical analysis framework. However, limited by the degree of sophistication and availability of data, the intrinsic motivational differences and specific types of differentiation in youth’s health insurance participation decision at the micro level need to be further researched. Simultaneously, urban–rural and individual health status differences are also important aspects of the internal and external motivations for young people’s health insurance choices that are worth exploring. It is essential to provide a more comprehensive and detailed theoretical basis and practical contribution to the multi-level social security system and to enhance young people’s confidence in development and sense of security.

## Data availability statement

The original contributions presented in the study are included in the article/supplementary material, further inquiries can be directed to the corresponding author.

## Author contributions

MC: Writing – original draft, Writing – review & editing. LW: Funding acquisition, Writing – review & editing.
